# Community Transmission of Severe Acute Respiratory Syndrome Coronavirus 2, Shenzhen, China, 2020

**DOI:** 10.3201/eid2606.200239

**Published:** 2020-06

**Authors:** Jiaye Liu, Xuejiao Liao, Shen Qian, Jing Yuan, Fuxiang Wang, Yingxia Liu, Zhaoqin Wang, Fu-Sheng Wang, Lei Liu, Zheng Zhang

**Affiliations:** Shenzhen Third People’s Hospital, Shenzhen, China (J. Liu, X. Liao, S. Qian, J. Yuan, F. Wang, Y. Liu, Z. Wang, L. Liu, Z. Zhang);; Southern University of Science and Technology, Shenzhen (Y. Liu, L. Liu, Z. Zhang);; The Fifth Medical Center of PLA General Hospital, Beijing, China (F.-S. Wang)

**Keywords:** 2019 novel coronavirus, community transmission, epidemiologic, severe respiratory syndrome coronavirus 2, SARS-CoV-2, COVID-19, viruses, Shenzhen, China, coronavirus, respiratory infections

## Abstract

Since early January 2020, after the outbreak of coronavirus infection in Wuhan, China, ≈365 confirmed cases have been reported in Shenzhen, China. The mode of community and intrafamily transmission is threatening residents in Shenzhen. Strategies to strengthen prevention and interruption of these transmissions should be urgently addressed.

In December 2019, an outbreak of a novel coronavirus infection (COVID-19), occurred in Wuhan, China (*1*). Although China launched an emergency response early in the outbreak, the infection rapidly spread to metropolitan areas in China and around the world. The growing number of cases suggests that the epidemic has continued to spread. Several research articles have reported the epidemiologic characteristics of the outbreak in Wuhan and Hubei Province (*1*–*4*); however, to our knowledge**,** an analysis of the epidemic in metropolis areas around Wuhan has not yet been reported. To predict the epidemic trend and guide control measures, especially in similar metropolitan areas, outbreak investigations are needed.

Shenzhen, a modern and international metropolitan city, is located in southern China (Appendix Figure 1) and has a population of 13 million persons, among which >1 million are from Hubei Province and >70,000 from Wuhan. After the first cluster of COVID-19 cases was confirmed in Shenzhen in early January 2020 (*5*), other cases spread within the city, involving all districts. To summarize the epidemiologic characteristics and provide updated information to aid in the development of control measures, we analyzed data for the first 365 laboratory-confirmed cases of COVID-19 in Shenzhen.

## The Cases

In early January 2020, a total of 24 days after the index COVID-19 case occurred in Wuhan, a familial cluster of COVID-19 case-patients who had traveled to Wuhan from December 29, 2019, through January 4, 2020, was identified in Shenzhen (*5*). Subsequently, more cases in the city were reported. Analysis of spatiotemporal dynamics indicated that the infection spread more broadly throughout the city (Figure; Appendix Figure 2). Since January 17, infections increased substantially, peaking January 22–30. The decline since January 30 is probably the result of underascertainment of cases with recent onset and delayed identification or reporting (Figure).

**Figure:**
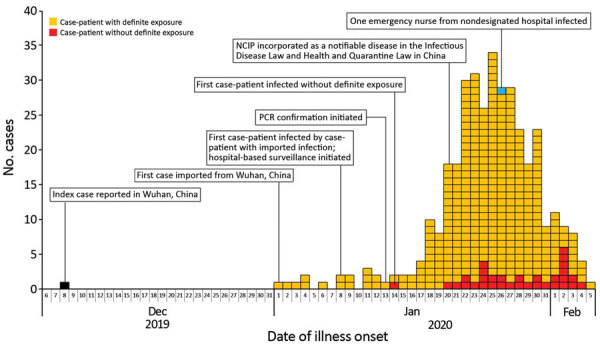
Onset of illness timeline for the first 365 confirmed COVID-19 case-patients in Shenzhen, China. The decline in incidence after January 30, 2020, probably resulted from delays in diagnosis and laboratory confirmation. All cases in this curve were confirmed. Hospital-based surveillance began January 8, 2020, for patients with suspected cases, defined by having a history of travel to Wuhan within the past 14 days, fever, and radiographic evidence of viral pneumonitis. PCR confirmation began January 13, 2020, and subsequently expanded the criteria for patients with suspected cases, defined by having typical clinical manifestations of COVID-19 and excluding infection caused by type A and B influenza and respiratory syncytial virus, regardless of travel history. NCIP, novel coronavirus–infected pneumonia (now called COVID-19).

Evaluation of the potential risk for local transmission will help determine whether patients with newly reported cases had definite exposure, defined by either having had definite contact with confirmed case-patients or having traveled to Wuhan or other cities in Hubei, or both, over the past 14 days. Overall, most (91%) cases that we report had definite exposure. On January 14, the first infected case-patient without definite exposure was reported in Shenzhen. Since January 20, growing numbers of cases without definite exposure were observed. Compared with the proportion before January 24, the proportion of case-patients without definite exposure was much higher from January 25 through February 5 (11% vs. 6%; p<0.001) and increased to 36% (12/33) on both January 31 and February 5. These data suggest an increasing risk for community transmission (Table 1; Figure).

**Table 1:** Characteristics of patients with severe acute respiratory syndrome coronavirus 2 infection in Shenzhen, China, as of February 5, 2020

Characteristic	Before Jan 24, n = 166	Jan 25–Feb 5, n = 199	p value
Median age (range), y	52 (1–81)	40 (1–86)	<0.001
Age group, no. (%)			<0.001
<15	4 (2)	26 (13)	
15–34	30 (18)	44 (22)	
35–54	60 (36)	70 (35)	
>55	72 (43)	59 (30)	
Patient sex, no. (%)			0.428
M	79 (48)	103 (52)	
F	87 (52)	96 (48)	
Exposure history, no. (%)			<0.001
Contact with confirmed case-patients	71 (43)	109 (55)	
Wuhan	78 (47)	42 (21)	
Cities other than Wuhan in Hubei Province	7 (4)	26 (13)	
No definite exposure	10 (6)	22 (11)	
First visited designated hospital, no. (%)	22 (13)	29 (15)	0.717
Days between illness onset and visiting hospital, median (range)	3 (0–15)	1 (0–9)	<0.001

We analyzed the clinical and epidemiologic characteristics of 365 persons with laboratory-confirmed cases in Shenzhen. The median case-patient age was 46 (range 1–86) years; 182 (50%) case-patients were male. To investigate the shift of the epidemic, we compared characteristics of case-patients during 2 periods: before January 24 (the Chinese Spring Festival) and after January 25 (until February 5). Because of delays between infection to illness onset or illness onset to confirmation, the following comparisons between the 2 periods might be biased because of misclassification. 

We found a sharply increasing proportion of infected children (from 2% before January 24 to 13% for January 25–February 5; p<0.001), implying that increased exposure for children and intrafamily transmission might contribute substantially to the epidemic. Although substantially higher after January 25, 2020, the proportion of infected children in our study before January 24, 2020, was similar to the proportions reported by Li et al (*1*) (0/425, based on cases as of January 22, 2020) and Guan et al. (W.J. Guan et al., unpub. data, https://doi.org/10.1101/2020.02.06.20020974) (9/1,099 as of January 29, 2020). The possible reasons for the discrepancy after January 25 might be the low proportion of children exposed early in the outbreak; early detection for children who had had close contact with persons with diagnosed or suspected cases after strict control measures were conducted comprehensively; and difficult identification of the relatively milder clinical signs and symptoms in young patients than in infected adults (*6*), especially in the setting of limited resources in the early phase of the outbreak in Wuhan.

We explored the incubation periods for 58 case-patients with definite exposure and detailed investigation information. The estimated mean incubation periods were 6.1 (range 1–16) days among 33 case-patients who had had close contact with symptomatic confirmed case-patients and 6.0 (range 1–15) days among 25 case-patients who had traveled to Wuhan and stayed <1 day over the previous 3 weeks (Table 2; Appendix Table 1). Estimated incubation periods were consistent with those previously reported (*1*). We analyzed the characteristics of 74 clusters involving 183 cases (2–6 cases/cluster). Among 12 clusters of single intracluster transmission cases, 15 case-patients were infected within 5.5 days of the mean interval between illness onset of the infector and illness onset of the infectee. Among 56 clusters of single co-exposure cases, the mean interval of symptom onset between the primary and second case-patient within a cluster was 3.1 days, and the mean interval of symptom onset between the primary and last case-patient within a cluster was 3.6 days (Appendix Table 2).

**Table 2:** Estimated incubation periods for severe acute respiratory syndrome coronavirus 2, stratified by exposure classification, Shenzhen, China, as of February 5, 2020

Exposure	No. patients	Mean, d	Median, d	Interquartile range, d	Range, d
Contact with confirmed symptomatic case-patient	33	6.1	5	3–8	1–16
Traveled to Wuhan and stayed <1 day	25	6.0	5	3–8	1–15
Total	58	6.0	5	3–8	1–16

With continuous implementation of strict control measures, we observed a shortened span (median days declining from 3 to 1; p<0.001) between illness onset and hospital visits for case-patients (Table 1). This finding may result from strict infection control management (e.g., early screening for suspected cases, monitoring for close-contact persons, and improved health consciousness of the general population).

To control the infection, confirmed case-patients should be separated and managed centrally; thus, the government has designated special hospitals to admit patients with suspected or confirmed cases. Nevertheless, as of February 5, to our knowledge, 1 case of a healthcare worker having been infected has been reported; an emergency nurse from a nondesignated hospital became ill on January 26, 2020, a total of 8 days after having been in close contact with a confirmed case-patient in the outpatient setting. We found that only 13%–15% of patients with confirmed cases went to the designated hospital first during the epidemic period. This finding means that a substantial number of case-patients visited >1 nondesignated hospital before they were admitted to the designated hospital, which increases the risk for nosocomial infection.

## Conclusions

Essential for the control of this extremely contagious disease are close monitoring and timely reporting of the epidemic to the public as well as evaluation of the current control strategy. On the basis of this epidemiologic analysis, we found that COVID-19 has become endemic to Shenzhen, China. We suspect that community transmission and intrafamily transmission have potentially become the new transmission modes in the city. Also, nosocomial infection and transmission might pose a potential risk for COVID-19 control. 

To control this outbreak in Shenzhen, maintaining basic and essential strategies is crucial. Early screening, diagnosis, isolation, and treatment are necessary to prevent further spread (*7*). Throughout the city, management of persons in close contact with persons with diagnosed and suspected cases, restriction of public activity, and use of personal protection measures should be continued. Strengthening effective and efficient measures, including but not limited to personal protection within families and communities with a high risk for exposure, will prevent and interrupt community and intrafamily transmission. To prevent nosocomial infection and transmission, a designated hospital should be the first choice for persons who had close contact with confirmed case-patients or who themselves have clinical signs indicative of COVID-19.

Appendix Detailed information for COVID-19 case-patients with regard to incubation period, clusters, and spatiotemporal dynamics, Shenzhen, China.
