# Supplementary material for: Community Transmission of Severe Acute Respiratory Syndrome Coronavirus 2, Shenzhen, China, 2020
Source: Emerg Infect Dis. 2020 Jun;26(6):1320–3. doi: 10.3201/eid2606.200239 (PMC7258448; doi:10.3201/eid2606.200239)
Supplement: Appendix — Detailed information for COVID-19 case-patients with regard to incubation period, clusters, and spatiotemporal dynamics, Shenzhen, China. [file 20-0239-Techapp-s1.pdf]

Article DOI: https://doi.org/10.3201/eid2606.200239

Community Transmission of Severe Acute Respiratory Syndrome Coronavirus 2, Shenzhen, China, 2020

Appendix

Appendix Table 1. The detailed case information for the analysis of the incubation period, stratified by exposure type

Number	Sex	Age (Years)	Onset of illness	Incubation period (Days)	Exposure
1	Female	53	2020/1/18	2	Travel to Wuhan and stay ≤1 day
2	Female	49	2020/1/18	7	Contact confirmed symptomatic cases
3	Male	73	2020/1/20	1	Travel to Wuhan and stay ≤1 day
4	Male	66	2020/1/20	4	Travel to Wuhan and stay ≤1 day
5	Male	53	2020/1/20	8	Travel to Wuhan and stay ≤1 day
6	Male	38	2020/1/21	2	Travel to Wuhan and stay ≤1 day
7	Female	39	2020/1/22	1	Contact confirmed symptomatic cases
8	Male	33	2020/1/22	6	Contact confirmed symptomatic cases
9	Male	25	2020/1/22	2	Travel to Wuhan and stay ≤1 day
10	Male	31	2020/1/22	6	Contact confirmed symptomatic cases
11	Female	78	2020/1/23	6	Contact confirmed symptomatic cases
12	Female	33	2020/1/23	1	Contact confirmed symptomatic cases
13	Female	32	2020/1/23	3	Travel to Wuhan and stay ≤1 day
14	Female	69	2020/1/24	5	Travel to Wuhan and stay ≤1 day
15	Male	61	2020/1/24	5	Contact confirmed symptomatic cases
16	Female	59	2020/1/24	1	Contact confirmed symptomatic cases
17	Male	38	2020/1/24	7	Travel to Wuhan and stay ≤1 day
18	Male	86	2020/1/25	2	Travel to Wuhan and stay ≤1 day
19	Female	62	2020/1/25	2	Contact confirmed symptomatic cases
20	Female	32	2020/1/25	6	Contact confirmed symptomatic cases
21	Male	41	2020/1/25	7	Travel to Wuhan and stay ≤1 day
22	Male	39	2020/1/25	2	Contact confirmed symptomatic cases
23	Female	31	2020/1/25	3	Travel to Wuhan and stay ≤1 day
24	Male	47	2020/1/25	2	Contact confirmed symptomatic cases
25	Male	48	2020/1/25	7	Travel to Wuhan and stay ≤1 day

Number	Sex	Age (Years)	Onset of illness	Incubation period (Days)	Exposure
26	Female	29	2020/1/26	5	Travel to Wuhan and stay ≤1 day
27	Female	33	2020/1/26	6	Travel to Wuhan and stay ≤1 day
28	Male	56	2020/1/26	1	Contact confirmed symptomatic cases
29	Male	41	2020/1/26	1	Contact confirmed symptomatic cases
30	Male	47	2020/1/26	7	Contact confirmed symptomatic cases
31	Male	65	2020/1/26	5	Contact confirmed symptomatic cases
32	Female	43	2020/1/26	5	Travel to Wuhan and stay ≤1 day
33	Female	25	2020/1/26	8	Contact confirmed symptomatic cases
34	Female	56	2020/1/26	4	Travel to Wuhan and stay ≤1 day
35	Female	35	2020/1/26	4	Contact confirmed symptomatic cases
36	Female	58	2020/1/27	4	Contact confirmed symptomatic cases
37	Female	38	2020/1/28	5	Contact confirmed symptomatic cases
38	Female	57	2020/1/28	10	Travel to Wuhan and stay ≤1 day
39	Female	50	2020/1/28	8	Contact confirmed symptomatic cases
40	Male	34	2020/1/28	10	Contact confirmed symptomatic cases
41	Male	40	2020/1/28	7	Travel to Wuhan and stay ≤1 day
42	Female	45	2020/1/29	5	Contact confirmed symptomatic cases
43	Female	40	2020/1/29	3	Contact confirmed symptomatic cases
44	Male	38	2020/1/29	5	Contact confirmed symptomatic cases
45	Male	45	2020/1/29	5	Travel to Wuhan and stay ≤1 day
46	Male	34	2020/1/29	10	Contact confirmed symptomatic cases
47	Female	7	2020/1/30	10	Travel to Wuhan and stay ≤1 day
48	Female	66	2020/1/30	10	Travel to Wuhan and stay ≤1 day
49	Female	65	2020/1/30	8	Contact confirmed symptomatic cases
50	Female	60	2020/1/31	9	Travel to Wuhan and stay ≤1 day
51	Female	58	2020/2/1	10	Travel to Wuhan and stay ≤1 day
52	Female	58	2020/2/2	5	Contact confirmed symptomatic cases
53	Male	31	2020/2/3	13	Contact confirmed symptomatic cases
54	Male	54	2020/2/3	15	Travel to Wuhan and stay ≤1 day
55	Male	2	2020/2/4	12	Contact confirmed symptomatic cases
56	Female	37	2020/2/4	14	Contact confirmed symptomatic cases
57	Female	37	2020/2/4	11	Contact confirmed symptomatic cases
58	Female	64	2020/2/5	16	Contact confirmed symptomatic cases

Appendix Table 2. The detailed case information for characteristic analysis of clusters

Cluster number	Case number in cluster	The relationship between primary case and other cases	Illness onset of primary cases in cluster	Interval of illness onset between the primary case and the second case	Interval of illness onset between the primary case and the last case	Co-exposure or intra-cluster transmission	The number of infectee in cluster	Interval of illness onset between infector and infectee
1	5	father-in-law, mother-in-law, son, mother	1-Jan	2	10	Both	1	4
2	3	husband, one close contact	4-Jan	8	21	Both	1	10
3	2	husband, daughter-in-law	12-Jan	3	10	Both	1	12
4	2	wife	11-Jan	9	9	Co-exposure		
5	2	husband	19-Jan	1	1	Co-exposure		
6	4	wife, granddaughter, co-mother-in-law	20-Jan	2	10	Co-exposure		
7	2	wife	16-Jan	0	0	Co-exposure		
8	2	wife	8-Jan	15	15	Co-exposure		
9	4	wife, son, daughter-in-law	20-Jan	2	4	Both	2	2, 4
10	2	wife	20-Jan	1	1	Co-exposure		
11	2	daughter	20-Jan	0	0	Co-exposure		
12	2	daughter	23-Jan	1	1	Co-exposure		
13	2	son	24-Jan	1	1	Intra-cluster transmission	1	1
14	4	friends	20-Jan	3	6	Intra-cluster transmission	3	3, 5, 6
15	5	wife, mother-in-law, daughter, wife, mother's sister	23-Jan	1	4	Co-exposure		
16	4	sister, mother, daughter	22-Jan	1	7	Both	1	6
17	3	daughter, son-in-law	24-Jan	2	2	Intra-cluster transmission	2	2, 2

Cluster number	Case number in cluster	The relationship between primary case and other cases	Illness onset of primary cases in cluster	Interval of illness onset between the primary case and the second case	Interval of illness onset between the primary case and the last case	Co-exposure or intra-cluster transmission	The number of infectee in cluster	Interval of illness onset between infector and infectee
18	5	wife，granddaughter1，granddaughter2，son	24-Jan	3	3	Both	1	0, 3
19	2	husband	24-Jan	2	2	Co-exposure		
20	2	father	21-Jan	6	6	Co-exposure		
21	2	daughter	25-Jan	0	0	Co-exposure		
22	2	husband	23-Jan	6	6	Intra-cluster transmission	1	6
23	4	wife，mother-in-law，father-in-law	19-Jan	6	7	Co-exposure		
24	2	husband	21-Jan	3	3	Co-exposure		
25	2	son	24-Jan	3	3	Co-exposure		
26	2	husband	23-Jan	2	2	Co-exposure		
27	2	son	18-Jan	6	6	Intra-cluster transmission	1	6
28	2	husband	27-Jan	3	3	Co-exposure		
29	2	wife	24-Jan	5	5	Co-exposure		
30	2	wife	23-Jan	1	1	Co-exposure		
31	2	daughter	26-Jan	3	3	Co-exposure		
32	2	mother	26-Jan	1	1	Co-exposure		
33	2	husband	26-Jan	1	1	Co-exposure		
34	2	wife	27-Jan	1	1	Co-exposure		
35	2	wife	30-Jan	0	0	Co-exposure		
36	4	son，grandson，husband	24-Jan	4	6	Co-exposure		
37	2	wife	18-Jan	4	4	Co-exposure		

Cluster number	Case number in cluster	The relationship between primary case and other cases	Illness onset of primary cases in cluster	Interval of illness onset between the primary case and the second case	Interval of illness onset between the primary case and the last case	Co-exposure or intra-cluster transmission	The number of infectee in cluster	Interval of illness onset between infector and infectee
38	2	mother	28-Jan	3	3	Co-exposure		
39	4	wife，daughter，son	22-Jan	6	8	Co-exposure		
40	2	wife	23-Jan	1	1	Co-exposure		
41	2	wife	26-Jan	3	3	Co-exposure		
42	3	mother，father	25-Jan	3	6	Co-exposure		
43	2	daughter	26-Jan	6	6	Co-exposure		
44	2	grandson	28-Jan	4	4	Co-exposure		
45	2	daughter	24-Jan	7	7	Co-exposure		
46	2	husband	24-Jan	3	3	Co-exposure		
47	2	husband	18-Jan	5	5	Co-exposure		
48	2	husband	23-Jan	3	3	Co-exposure		
49	3	son，daughter-in-law	22-Jan	8	8	Co-exposure		
50	2	daughter	27-Jan	2	2	Co-exposure		
51	2	daughter-in-law	24-Jan	4	4	Intra-cluster transmission	1	4
52	2	father	27-Jan	2	2	Co-exposure		
53	2	daughter	25-Jan	4	4	Co-exposure		
54	2	daughter-in-law	22-Jan	1	1	Co-exposure		
55	2	daughter	17-Jan	6	6	Intra-cluster transmission	1	6
56	3	wife，daughter	1-Feb	1	1	Co-exposure		
57	2	wife	26-Jan	4	4	Intra-cluster transmission	1	4
58	2	husband	25-Jan	1	1	Co-exposure		

Cluster number	Case number in cluster	The relationship between primary case and other cases	Illness onset of primary cases in cluster	Interval of illness onset between the primary case and the second case	Interval of illness onset between the primary case and the last case	Co-exposure or intra-cluster transmission	The number of infectee in cluster	Interval of illness onset between infector and infectee
59	3	girlfriend, father	3-Feb	1	1	Co-exposure		
60	2	wife	27-Jan	0	0	Co-exposure		
61	6	husband, daughter1, daughter2, father, mother	28-Jan	2	5	Co-exposure		
62	2	son	24-Jan	10	10	Intra-cluster transmission	1	10
63	2	husband	28-Jan	4	4	Co-exposure		
64	2	wife	27-Jan	0	0	Co-exposure		
65	2	son	27-Jan	9	9	Co-exposure		
66	2	son	26-Jan	0	0	Co-exposure		
67	2	daughter-in-law	19-Jan	16	16	Intra-cluster transmission	1	16
68	2	wife	29-Jan	7	7	Intra-cluster transmission	1	7
69	3	wife, daughter	27-Jan	7	9	Co-exposure		
70	2	wife	25-Jan	10	10	Co-exposure		
71	2	son	31-Jan	1	1	Co-exposure		
72	3	son, wife	3-Feb	2	2	Co-exposure		
73	2	sister	3-Feb	2	2	Co-exposure		
74	2	daughter	30-Jan	4	4	Intra-cluster transmission	1	4

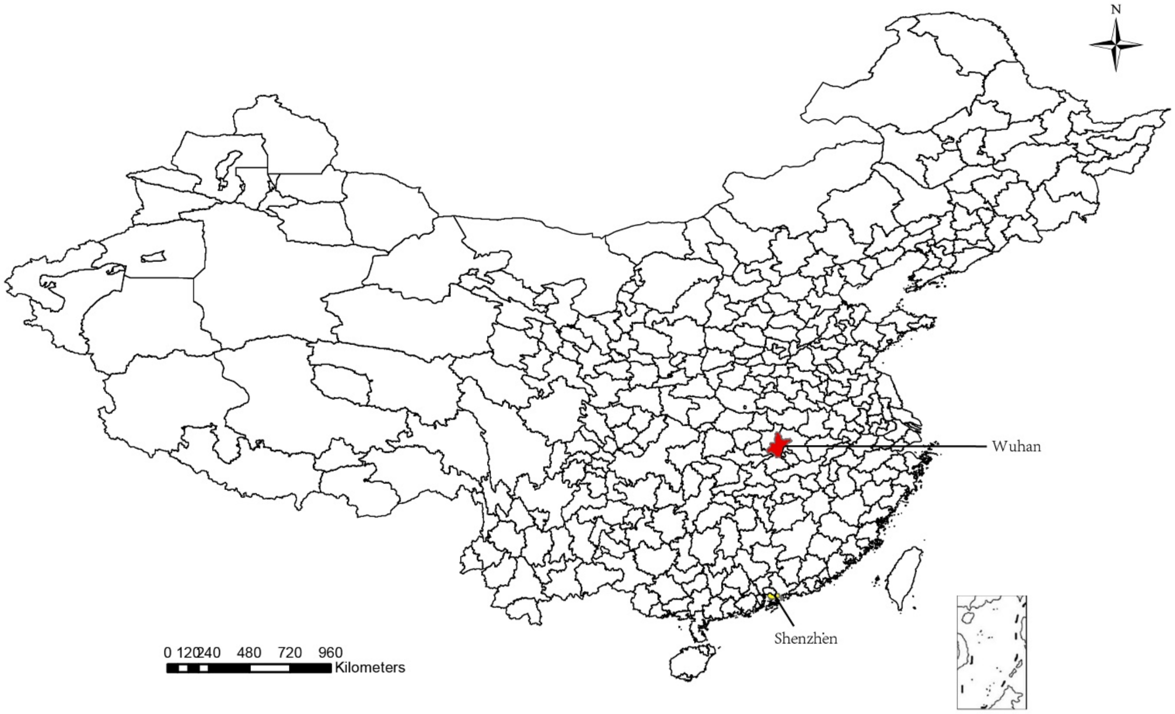

Appendix Figure 1. The location of Wuhan city and Shenzhen city, China.

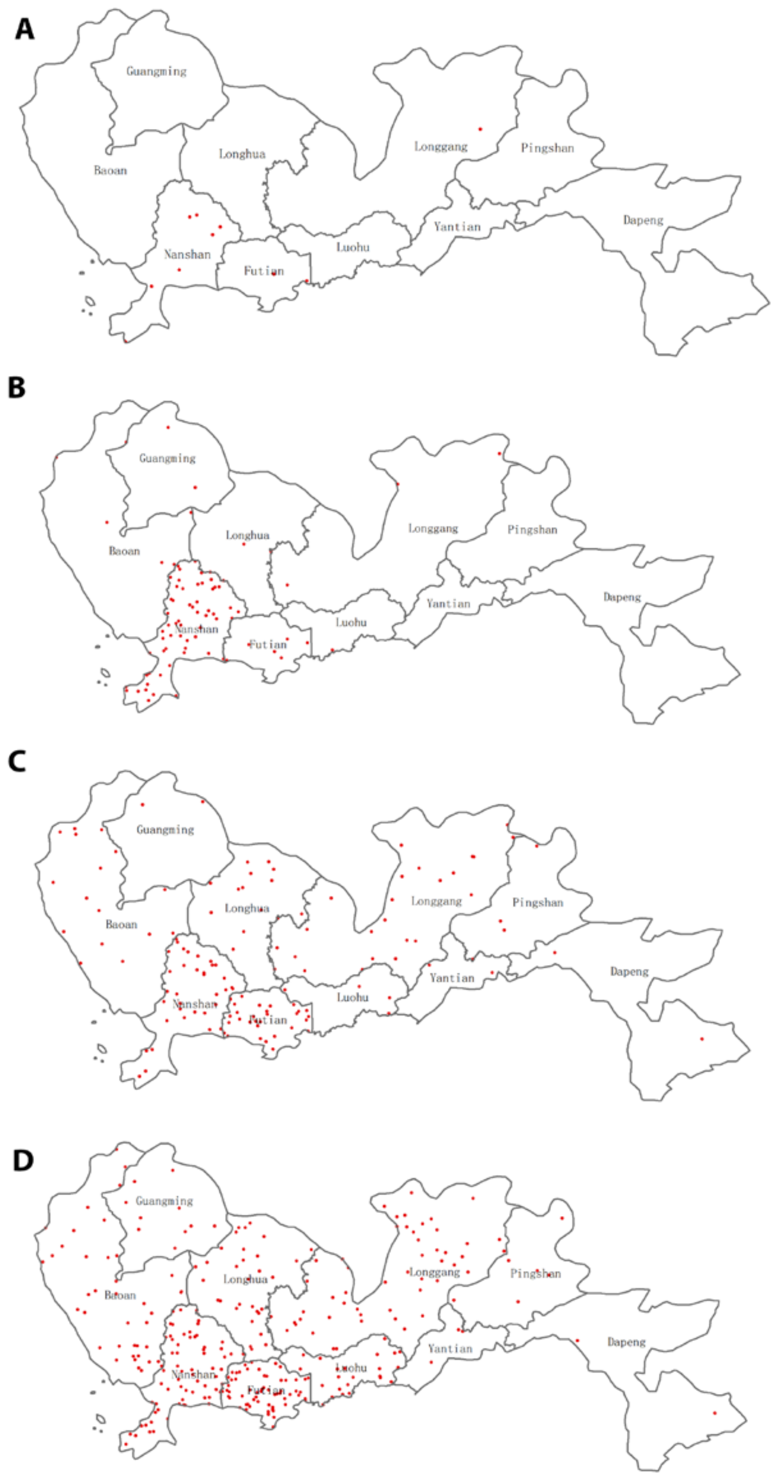

Appendix Figure 2. Spatiotemporal dynamics of the first 365 confirmed cases of 2019-nCoV in Shenzhen, China. The geographic distribution of cases was presented based on the onset of illness as of January 10 (A), January 20 (B), January 31 (C), and February 5 (D).
